# Supplementary material for: Transcriptome Analysis Reveals Vimentin-Induced Disruption of Cell–Cell Associations Augments Breast Cancer Cell Migration
Source: Cells. 2022 Dec 13;11(24):4035. doi: 10.3390/cells11244035 (PMC9776984; doi:10.3390/cells11244035)
Supplement: Supplementary file 1 [file cells-11-04035-s001.zip › DEGs and enrichment analysis.zip!/DEGs and enrichment analysis/WT_VIMvsVector KEGG/UP/WT_VIMvsVector_UP.pathway_map.zip!/WT_VIMvsVector_UP.pathway_map/WT_VIMvsVector_up_KEGGenrich.xls_rendered_html_detail.html]

Pathway Enrichment


  

# The most enriched pathway terms

Statistic method: hypergeometric test

FDR correction method: Benjamini and Hochberg

| Term | Description | Sample number | Background number | P-value | Corrected P-value | Gene\_id | Gene\_name | KEGG\_ID |
| --- | --- | --- | --- | --- | --- | --- | --- | --- |
| hsa03030 | DNA replication | 9\|135 | 41\|9228 | 5.53319401252324e-09 | 1.13430477256726e-06 | ENSG00000073111 ENSG00000100297 ENSG00000166508 ENSG00000168496 ENSG00000049541 ENSG00000062822 ENSG00000106628 ENSG00000014138 ENSG00000198056 | MCM2 MCM5 MCM7 FEN1 RFC2 POLD1 POLD2 POLA2 PRIM1 | hsa:4171 hsa:4174 hsa:4176 hsa:2237 hsa:5982 hsa:5424 hsa:5425 hsa:23649 hsa:5557 |
| hsa04110 | Cell cycle | 15\|135 | 163\|9228 | 1.3122613615519e-08 | 1.3450678955907e-06 | novel.507 ENSG00000093009 ENSG00000127564 ENSG00000166851 ENSG00000117399 ENSG00000073111 ENSG00000085840 ENSG00000135476 ENSG00000100297 ENSG00000166508 ENSG00000101412 ENSG00000145386 ENSG00000164045 ENSG00000157456 ENSG00000116670 | - CDC45 PKMYT1 PLK1 CDC20 MCM2 ORC1 ESPL1 MCM5 MCM7 E2F1 CCNA2 CDC25A CCNB2 MAD2L2 | hsa:1870 hsa:8318 hsa:9088 hsa:5347 hsa:991 hsa:4171 hsa:4998 hsa:9700 hsa:4174 hsa:4176 hsa:1869 hsa:890 hsa:993 hsa:9133 hsa:10459 |
| hsa04914 | Progesterone-mediated oocyte maturation | 8\|135 | 120\|9228 | 0.000362184463137663 | 0.0247492716477403 | ENSG00000082175 ENSG00000127564 ENSG00000166851 ENSG00000145386 ENSG00000164045 ENSG00000164742 ENSG00000157456 ENSG00000116670 | PGR PKMYT1 PLK1 CCNA2 CDC25A ADCY1 CCNB2 MAD2L2 | hsa:5241 hsa:9088 hsa:5347 hsa:890 hsa:993 hsa:107 hsa:9133 hsa:10459 |
| hsa04114 | Oocyte meiosis | 8\|135 | 171\|9228 | 0.00355770126547273 | 0.182332189855478 | ENSG00000082175 ENSG00000127564 ENSG00000166851 ENSG00000117399 ENSG00000135476 ENSG00000164742 ENSG00000157456 ENSG00000116670 | PGR PKMYT1 PLK1 CDC20 ESPL1 ADCY1 CCNB2 MAD2L2 | hsa:5241 hsa:9088 hsa:5347 hsa:991 hsa:9700 hsa:107 hsa:9133 hsa:10459 |
| hsa05166 | Human T-cell leukemia virus 1 infection | 10\|135 | 261\|9228 | 0.00488891448082025 | 0.18841248879852 | novel.507 ENSG00000117399 ENSG00000135476 ENSG00000101412 ENSG00000145386 ENSG00000164742 ENSG00000117394 novel.600 ENSG00000164362 ENSG00000157456 | - CDC20 ESPL1 E2F1 CCNA2 ADCY1 SLC2A1 - TERT CCNB2 | hsa:1870 hsa:991 hsa:9700 hsa:1869 hsa:890 hsa:107 hsa:6513 hsa:1388 hsa:7015 hsa:9133 |
| hsa05206 | MicroRNAs in cancer | 8\|135 | 185\|9228 | 0.00570697859648851 | 0.18841248879852 | ENSG00000026025 novel.507 ENSG00000101412 ENSG00000171791 ENSG00000146670 ENSG00000164045 ENSG00000156103 ENSG00000088305 | VIM - E2F1 BCL2 CDCA5 CDC25A MMP16 DNMT3B | hsa:7431 hsa:1870 hsa:1869 hsa:596 hsa:113130 hsa:993 hsa:4325 hsa:1789 |
| hsa03410 | Base excision repair | 4\|135 | 51\|9228 | 0.00643359717848603 | 0.18841248879852 | ENSG00000168496 ENSG00000065057 ENSG00000062822 ENSG00000106628 | FEN1 NTHL1 POLD1 POLD2 | hsa:2237 hsa:4913 hsa:5424 hsa:5425 |
| hsa04115 | p53 signaling pathway | 5\|135 | 89\|9228 | 0.00968481803729423 | 0.248173462205665 | ENSG00000177519 ENSG00000171791 ENSG00000075218 ENSG00000184990 ENSG00000157456 | RPRM BCL2 GTSE1 SIVA1 CCNB2 | hsa:56475 hsa:596 hsa:51512 hsa:10572 hsa:9133 |
| hsa01522 | Endocrine resistance | 5\|135 | 99\|9228 | 0.0148739999845145 | 0.296293338063755 | ENSG00000164850 novel.507 ENSG00000101412 ENSG00000171791 ENSG00000164742 | GPER1 - E2F1 BCL2 ADCY1 | hsa:2852 hsa:1870 hsa:1869 hsa:596 hsa:107 |
| hsa05161 | Hepatitis B | 7\|135 | 179\|9228 | 0.0159309939364494 | 0.296293338063755 | novel.507 ENSG00000179388 ENSG00000089685 ENSG00000101412 ENSG00000171791 ENSG00000145386 novel.600 | - EGR3 BIRC5 E2F1 BCL2 CCNA2 - | hsa:1870 hsa:1960 hsa:332 hsa:1869 hsa:596 hsa:890 hsa:1388 |
| hsa04915 | Estrogen signaling pathway | 7\|135 | 180\|9228 | 0.0163794029006418 | 0.296293338063755 | ENSG00000082175 ENSG00000164850 ENSG00000160182 ENSG00000131759 ENSG00000171791 ENSG00000164742 novel.600 | PGR GPER1 TFF1 RARA BCL2 ADCY1 - | hsa:5241 hsa:2852 hsa:7031 hsa:5914 hsa:596 hsa:107 hsa:1388 |
| hsa00240 | Pyrimidine metabolism | 4\|135 | 68\|9228 | 0.0173440002769027 | 0.296293338063755 | ENSG00000167900 ENSG00000128951 ENSG00000176890 ENSG00000168393 | TK1 DUT TYMS DTYMK | hsa:7083 hsa:1854 hsa:7298 hsa:1841 |
| hsa03430 | Mismatch repair | 3\|135 | 39\|9228 | 0.0190665974477478 | 0.300665575137561 | ENSG00000049541 ENSG00000062822 ENSG00000106628 | RFC2 POLD1 POLD2 | hsa:5982 hsa:5424 hsa:5425 |
| hsa04928 | Parathyroid hormone synthesis, secretion and action | 5\|135 | 113\|9228 | 0.024863620399118 | 0.364074441558514 | ENSG00000171791 ENSG00000164742 novel.600 ENSG00000109062 ENSG00000156103 | BCL2 ADCY1 - SLC9A3R1 MMP16 | hsa:596 hsa:107 hsa:1388 hsa:9368 hsa:4325 |
| hsa04911 | Insulin secretion | 4\|135 | 78\|9228 | 0.0271918059515094 | 0.371621348003961 | ENSG00000104783 ENSG00000164742 ENSG00000117394 novel.600 | KCNN4 ADCY1 SLC2A1 - | hsa:3783 hsa:107 hsa:6513 hsa:1388 |
| hsa04218 | Cellular senescence | 7\|135 | 204\|9228 | 0.0300006766285159 | 0.373917906451761 | novel.507 ENSG00000111206 ENSG00000101057 ENSG00000101412 ENSG00000145386 ENSG00000164045 ENSG00000157456 | - FOXM1 MYBL2 E2F1 CCNA2 CDC25A CCNB2 | hsa:1870 hsa:2305 hsa:4605 hsa:1869 hsa:890 hsa:993 hsa:9133 |
| hsa05222 | Small cell lung cancer | 5\|135 | 121\|9228 | 0.0321407999083554 | 0.373917906451761 | novel.507 ENSG00000005961 ENSG00000101412 ENSG00000171791 ENSG00000173207 | - ITGA2B E2F1 BCL2 CKS1B | hsa:1870 hsa:3674 hsa:1869 hsa:596 hsa:1163 |
| hsa03440 | Homologous recombination | 3\|135 | 48\|9228 | 0.0328318161762522 | 0.373917906451761 | ENSG00000154920 ENSG00000062822 ENSG00000106628 | EME1 POLD1 POLD2 | hsa:146956 hsa:5424 hsa:5425 |
| hsa04974 | Protein digestion and absorption | 4\|135 | 85\|9228 | 0.0357292229644087 | 0.385499510931778 | ENSG00000092068 ENSG00000164626 ENSG00000104783 ENSG00000168542 | SLC7A8 KCNK5 KCNN4 COL3A1 | hsa:23428 hsa:8645 hsa:3783 hsa:1281 |
| hsa04080 | Neuroactive ligand-receptor interaction | 7\|135 | 219\|9228 | 0.04158583237195 | 0.426254781812487 | ENSG00000164128 ENSG00000187730 ENSG00000109255 ENSG00000168539 ENSG00000069482 ENSG00000171631 ENSG00000180739 | NPY1R GABRD NMU CHRM1 GAL P2RY6 S1PR5 | hsa:4886 hsa:2563 hsa:10874 hsa:1128 hsa:51083 hsa:5031 hsa:53637 |
| hsa03420 | Nucleotide excision repair | 3\|135 | 56\|9228 | 0.0484070047226755 | 0.472544569911832 | ENSG00000049541 ENSG00000062822 ENSG00000106628 | RFC2 POLD1 POLD2 | hsa:5982 hsa:5424 hsa:5425 |
| hsa00330 | Arginine and proline metabolism | 3\|135 | 58\|9228 | 0.0527753205222041 | 0.49177003213872 | ENSG00000104524 ENSG00000166165 ENSG00000137124 | PYCR3 CKB ALDH1B1 | hsa:65263 hsa:1152 hsa:219 |
| hsa05224 | Breast cancer | 5\|135 | 147\|9228 | 0.0643001640829624 | 0.526074980633715 | ENSG00000082175 ENSG00000135547 novel.507 ENSG00000101412 ENSG00000037280 | PGR HEY2 - E2F1 FLT4 | hsa:5241 hsa:23493 hsa:1870 hsa:1869 hsa:2324 |
| hsa00270 | Cysteine and methionine metabolism | 3\|135 | 63\|9228 | 0.0644918074837717 | 0.526074980633715 | ENSG00000151224 ENSG00000088305 ENSG00000092621 | MAT1A DNMT3B PHGDH | hsa:4143 hsa:1789 hsa:26227 |
| hsa04540 | Gap junction | 4\|135 | 104\|9228 | 0.0658883714485255 | 0.526074980633715 | ENSG00000214222 ENSG00000123416 ENSG00000164742 ENSG00000104833 | TUBBP2 TUBA1B ADCY1 TUBB4A | hsa:203068 hsa:10376 hsa:107 hsa:10382 |
| hsa00100 | Steroid biosynthesis | 2\|135 | 29\|9228 | 0.0667217048608614 | 0.526074980633715 | ENSG00000167780 ENSG00000147155 | SOAT2 EBP | hsa:8435 hsa:10682 |
| hsa04725 | Cholinergic synapse | 4\|135 | 108\|9228 | 0.073520024622127 | 0.558207594353186 | ENSG00000127588 ENSG00000168539 ENSG00000171791 ENSG00000164742 | GNG13 CHRM1 BCL2 ADCY1 | hsa:51764 hsa:1128 hsa:596 hsa:107 |
| hsa05034 | Alcoholism | 6\|135 | 205\|9228 | 0.0800631951770439 | 0.571206888221669 | ENSG00000127588 ENSG00000184260 ENSG00000124635 ENSG00000180176 novel.600 ENSG00000218690 | GNG13 HIST2H2AC HIST1H2BJ TH - HIST1H2APS4 | hsa:51764 hsa:8338 hsa:8970 hsa:7054 hsa:1388 hsa:8336 |
| hsa05032 | Morphine addiction | 3\|135 | 70\|9228 | 0.0827088885002286 | 0.571206888221669 | ENSG00000127588 ENSG00000187730 ENSG00000164742 | GNG13 GABRD ADCY1 | hsa:51764 hsa:2563 hsa:107 |
| hsa01523 | Antifolate resistance | 2\|135 | 33\|9228 | 0.0835912519348784 | 0.571206888221669 | ENSG00000176890 ENSG00000173638 | TYMS SLC19A1 | hsa:7298 hsa:6573 |
| hsa04727 | GABAergic synapse | 3\|135 | 78\|9228 | 0.1058983267515 | 0.700295386582503 | ENSG00000127588 ENSG00000187730 ENSG00000164742 | GNG13 GABRD ADCY1 | hsa:51764 hsa:2563 hsa:107 |
| hsa04926 | Relaxin signaling pathway | 4\|135 | 133\|9228 | 0.130477249093375 | 0.821442387541214 | ENSG00000127588 ENSG00000164742 novel.600 ENSG00000168542 | GNG13 ADCY1 - COL3A1 | hsa:51764 hsa:107 hsa:1388 hsa:1281 |
| hsa00430 | Taurine and hypotaurine metabolism | 1\|135 | 10\|9228 | 0.137091815465662 | 0.821442387541214 | ENSG00000167741 | GGT6 | hsa:124975 |
| hsa00920 | Sulfur metabolism | 1\|135 | 10\|9228 | 0.137091815465662 | 0.821442387541214 | ENSG00000143416 | SELENBP1 | hsa:8991 |
| hsa04742 | Taste transduction | 2\|135 | 45\|9228 | 0.140285335631349 | 0.821442387541214 | ENSG00000127588 ENSG00000164626 | GNG13 KCNK5 | hsa:51764 hsa:8645 |
| hsa04215 | Apoptosis - multiple species | 2\|135 | 46\|9228 | 0.145324449385103 | 0.821442387541214 | ENSG00000089685 ENSG00000171791 | BIRC5 BCL2 | hsa:332 hsa:596 |
| hsa05030 | Cocaine addiction | 2\|135 | 47\|9228 | 0.150400930559514 | 0.821442387541214 | ENSG00000180176 novel.600 | TH - | hsa:7054 hsa:1388 |
| hsa05219 | Bladder cancer | 2\|135 | 48\|9228 | 0.155512631184874 | 0.821442387541214 | novel.507 ENSG00000101412 | - E2F1 | hsa:1870 hsa:1869 |
| hsa05163 | Human cytomegalovirus infection | 6\|135 | 251\|9228 | 0.16189248735959 | 0.821442387541214 | ENSG00000107562 novel.507 ENSG00000127588 ENSG00000101412 ENSG00000164742 novel.600 | CXCL12 - GNG13 E2F1 ADCY1 - | hsa:6387 hsa:1870 hsa:51764 hsa:1869 hsa:107 hsa:1388 |
| hsa05226 | Gastric cancer | 4\|135 | 148\|9228 | 0.171290753034385 | 0.821442387541214 | novel.507 ENSG00000101412 ENSG00000171791 ENSG00000164362 | - E2F1 BCL2 TERT | hsa:1870 hsa:1869 hsa:596 hsa:7015 |
| hsa00600 | Sphingolipid metabolism | 2\|135 | 52\|9228 | 0.17627053541793 | 0.821442387541214 | ENSG00000168350 ENSG00000102393 | DEGS2 GLA | hsa:123099 hsa:2717 |
| hsa04360 | Axon guidance | 5\|135 | 204\|9228 | 0.178916790101534 | 0.821442387541214 | ENSG00000107562 ENSG00000157851 ENSG00000167680 ENSG00000065320 ENSG00000044524 | CXCL12 DPYSL5 SEMA6B NTN1 EPHA3 | hsa:6387 hsa:56896 hsa:10501 hsa:9423 hsa:2042 |
| hsa04151 | PI3K-Akt signaling pathway | 8\|135 | 376\|9228 | 0.185278715029917 | 0.821442387541214 | ENSG00000005961 ENSG00000160867 ENSG00000127588 ENSG00000168539 ENSG00000171791 novel.600 ENSG00000037280 ENSG00000118513 | ITGA2B FGFR4 GNG13 CHRM1 BCL2 - FLT4 MYB | hsa:3674 hsa:2264 hsa:51764 hsa:1128 hsa:596 hsa:1388 hsa:2324 hsa:4602 |
| hsa04950 | Maturity onset diabetes of the young | 1\|135 | 14\|9228 | 0.186550067872609 | 0.821442387541214 | ENSG00000116833 | NR5A2 | hsa:2494 |
| hsa00260 | Glycine, serine and threonine metabolism | 2\|135 | 54\|9228 | 0.186808822651154 | 0.821442387541214 | ENSG00000123453 ENSG00000092621 | SARDH PHGDH | hsa:1757 hsa:26227 |
| hsa04923 | Regulation of lipolysis in adipocytes | 2\|135 | 55\|9228 | 0.192111315168113 | 0.821442387541214 | ENSG00000164128 ENSG00000164742 | NPY1R ADCY1 | hsa:4886 hsa:107 |
| hsa00603 | Glycosphingolipid biosynthesis - globo and isoglobo series | 1\|135 | 16\|9228 | 0.210213433240097 | 0.821442387541214 | ENSG00000102393 | GLA | hsa:2717 |
| hsa00604 | Glycosphingolipid biosynthesis - ganglio series | 1\|135 | 17\|9228 | 0.221787595909722 | 0.821442387541214 | ENSG00000135454 | B4GALNT1 | hsa:2583 |
| hsa03450 | Non-homologous end-joining | 1\|135 | 17\|9228 | 0.221787595909722 | 0.821442387541214 | ENSG00000168496 | FEN1 | hsa:2237 |
| hsa04934 | Cushing syndrome | 4\|135 | 166\|9228 | 0.225219630098023 | 0.821442387541214 | novel.507 ENSG00000101412 ENSG00000164742 novel.600 | - E2F1 ADCY1 - | hsa:1870 hsa:1869 hsa:107 hsa:1388 |
| hsa00480 | Glutathione metabolism | 2\|135 | 62\|9228 | 0.229693546543149 | 0.821442387541214 | ENSG00000167741 ENSG00000166825 | GGT6 ANPEP | hsa:124975 hsa:290 |
| hsa05033 | Nicotine addiction | 1\|135 | 18\|9228 | 0.233193379706508 | 0.821442387541214 | ENSG00000187730 | GABRD | hsa:2563 |
| hsa05215 | Prostate cancer | 3\|135 | 115\|9228 | 0.23695212358446 | 0.821442387541214 | novel.507 ENSG00000101412 ENSG00000171791 | - E2F1 BCL2 | hsa:1870 hsa:1869 hsa:596 |
| hsa04614 | Renin-angiotensin system | 1\|135 | 19\|9228 | 0.244433216160321 | 0.821442387541214 | ENSG00000166825 | ANPEP | hsa:290 |
| hsa04927 | Cortisol synthesis and secretion | 2\|135 | 65\|9228 | 0.245964007893934 | 0.821442387541214 | ENSG00000164742 novel.600 | ADCY1 - | hsa:107 hsa:1388 |
| hsa05031 | Amphetamine addiction | 2\|135 | 65\|9228 | 0.245964007893934 | 0.821442387541214 | ENSG00000180176 novel.600 | TH - | hsa:7054 hsa:1388 |
| hsa05169 | Epstein-Barr virus infection | 5\|135 | 230\|9228 | 0.246877079217675 | 0.821442387541214 | ENSG00000026025 novel.507 ENSG00000101412 ENSG00000171791 ENSG00000145386 | VIM - E2F1 BCL2 CCNA2 | hsa:7431 hsa:1870 hsa:1869 hsa:596 hsa:890 |
| hsa01230 | Biosynthesis of amino acids | 3\|135 | 118\|9228 | 0.248683510026468 | 0.821442387541214 | ENSG00000104524 ENSG00000151224 ENSG00000092621 | PYCR3 MAT1A PHGDH | hsa:65263 hsa:4143 hsa:26227 |
| hsa00910 | Nitrogen metabolism | 1\|135 | 20\|9228 | 0.255509501947959 | 0.821442387541214 | ENSG00000074410 | CA12 | hsa:771 |
| hsa00561 | Glycerolipid metabolism | 2\|135 | 67\|9228 | 0.256837241485664 | 0.821442387541214 | ENSG00000137124 ENSG00000102393 | ALDH1B1 GLA | hsa:219 hsa:2717 |
| hsa04640 | Hematopoietic cell lineage | 2\|135 | 67\|9228 | 0.256837241485664 | 0.821442387541214 | ENSG00000005961 ENSG00000166825 | ITGA2B ANPEP | hsa:3674 hsa:290 |
| hsa04929 | GnRH secretion | 2\|135 | 68\|9228 | 0.262277859142159 | 0.821442387541214 | ENSG00000164850 ENSG00000104783 | GPER1 KCNN4 | hsa:2852 hsa:3783 |
| hsa00053 | Ascorbate and aldarate metabolism | 1\|135 | 21\|9228 | 0.266424599388991 | 0.821442387541214 | ENSG00000137124 | ALDH1B1 | hsa:219 |
| hsa04977 | Vitamin digestion and absorption | 1\|135 | 21\|9228 | 0.266424599388991 | 0.821442387541214 | ENSG00000173638 | SLC19A1 | hsa:6573 |
| hsa05322 | Systemic lupus erythematosus | 3\|135 | 123\|9228 | 0.268424788743405 | 0.821442387541214 | ENSG00000184260 ENSG00000124635 ENSG00000218690 | HIST2H2AC HIST1H2BJ HIST1H2APS4 | hsa:8338 hsa:8970 hsa:8336 |
| hsa05218 | Melanoma | 2\|135 | 70\|9228 | 0.273160993861341 | 0.821442387541214 | novel.507 ENSG00000101412 | - E2F1 | hsa:1870 hsa:1869 |
| hsa04015 | Rap1 signaling pathway | 5\|135 | 240\|9228 | 0.274673652362061 | 0.821442387541214 | ENSG00000005961 ENSG00000160867 ENSG00000164742 ENSG00000037280 ENSG00000233328 | ITGA2B FGFR4 ADCY1 FLT4 PFN1P1 | hsa:3674 hsa:2264 hsa:107 hsa:2324 hsa:5216 |
| hsa05221 | Acute myeloid leukemia | 2\|135 | 72\|9228 | 0.284038926154079 | 0.821442387541214 | ENSG00000131759 ENSG00000145386 | RARA CCNA2 | hsa:5914 hsa:890 |
| hsa04918 | Thyroid hormone synthesis | 2\|135 | 73\|9228 | 0.28947316542375 | 0.821442387541214 | ENSG00000164742 novel.600 | ADCY1 - | hsa:107 hsa:1388 |
| hsa04976 | Bile secretion | 2\|135 | 73\|9228 | 0.28947316542375 | 0.821442387541214 | ENSG00000164742 ENSG00000117394 | ADCY1 SLC2A1 | hsa:107 hsa:6513 |
| hsa03460 | Fanconi anemia pathway | 2\|135 | 74\|9228 | 0.294902837413011 | 0.821442387541214 | ENSG00000154920 ENSG00000221829 | EME1 FANCG | hsa:146956 hsa:2189 |
| hsa04071 | Sphingolipid signaling pathway | 3\|135 | 132\|9228 | 0.304364226523505 | 0.821442387541214 | ENSG00000168350 ENSG00000171791 ENSG00000180739 | DEGS2 BCL2 S1PR5 | hsa:123099 hsa:596 hsa:53637 |
| hsa04728 | Dopaminergic synapse | 3\|135 | 133\|9228 | 0.308377002925738 | 0.821442387541214 | ENSG00000127588 ENSG00000180176 novel.600 | GNG13 TH - | hsa:51764 hsa:7054 hsa:1388 |
| hsa00340 | Histidine metabolism | 1\|135 | 25\|9228 | 0.308519176758367 | 0.821442387541214 | ENSG00000137124 | ALDH1B1 | hsa:219 |
| hsa04145 | Phagosome | 4\|135 | 192\|9228 | 0.309215458839249 | 0.821442387541214 | ENSG00000214222 ENSG00000123416 ENSG00000102879 ENSG00000104833 | TUBBP2 TUBA1B CORO1A TUBB4A | hsa:203068 hsa:10376 hsa:11151 hsa:10382 |
| hsa04917 | Prolactin signaling pathway | 2\|135 | 77\|9228 | 0.311154372030367 | 0.821442387541214 | ENSG00000114737 ENSG00000180176 | CISH TH | hsa:1154 hsa:7054 |
| hsa05223 | Non-small cell lung cancer | 2\|135 | 77\|9228 | 0.311154372030367 | 0.821442387541214 | novel.507 ENSG00000101412 | - E2F1 | hsa:1870 hsa:1869 |
| hsa05165 | Human papillomavirus infection | 7\|135 | 377\|9228 | 0.31410899305633 | 0.821442387541214 | ENSG00000135547 ENSG00000005961 ENSG00000101412 ENSG00000145386 ENSG00000131462 ENSG00000109062 ENSG00000164362 | HEY2 ITGA2B E2F1 CCNA2 TUBG1 SLC9A3R1 TERT | hsa:23493 hsa:3674 hsa:1869 hsa:890 hsa:7283 hsa:9368 hsa:7015 |
| hsa04970 | Salivary secretion | 2\|135 | 78\|9228 | 0.316555846906126 | 0.821442387541214 | ENSG00000104783 ENSG00000164742 | KCNN4 ADCY1 | hsa:3783 hsa:107 |
| hsa04810 | Regulation of actin cytoskeleton | 5\|135 | 257\|9228 | 0.323273518167854 | 0.828388390305125 | ENSG00000107562 ENSG00000005961 ENSG00000160867 ENSG00000168539 ENSG00000233328 | CXCL12 ITGA2B FGFR4 CHRM1 PFN1P1 | hsa:6387 hsa:3674 hsa:2264 hsa:1128 hsa:5216 |
| hsa00670 | One carbon pool by folate | 1\|135 | 28\|9228 | 0.338508450286589 | 0.832362184316392 | ENSG00000176890 | TYMS | hsa:7298 |
| hsa00790 | Folate biosynthesis | 1\|135 | 28\|9228 | 0.338508450286589 | 0.832362184316392 | ENSG00000180176 | TH | hsa:7054 |
| hsa04611 | Platelet activation | 3\|135 | 141\|9228 | 0.340517982748618 | 0.832362184316392 | ENSG00000005961 ENSG00000164742 ENSG00000168542 | ITGA2B ADCY1 COL3A1 | hsa:3674 hsa:107 hsa:1281 |
| hsa00052 | Galactose metabolism | 1\|135 | 29\|9228 | 0.348215119766079 | 0.832362184316392 | ENSG00000102393 | GLA | hsa:2717 |
| hsa04672 | Intestinal immune network for IgA production | 1\|135 | 29\|9228 | 0.348215119766079 | 0.832362184316392 | ENSG00000107562 | CXCL12 | hsa:6387 |
| hsa00310 | Lysine degradation | 2\|135 | 85\|9228 | 0.354056180975627 | 0.832362184316392 | ENSG00000101945 ENSG00000137124 | SUV39H1 ALDH1B1 | hsa:6839 hsa:219 |
| hsa05214 | Glioma | 2\|135 | 87\|9228 | 0.364647173738214 | 0.832362184316392 | novel.507 ENSG00000101412 | - E2F1 | hsa:1870 hsa:1869 |
| hsa04630 | JAK-STAT signaling pathway | 3\|135 | 148\|9228 | 0.368577022847428 | 0.832362184316392 | ENSG00000114737 ENSG00000171791 ENSG00000104998 | CISH BCL2 IL27RA | hsa:1154 hsa:596 hsa:9466 |
| hsa01524 | Platinum drug resistance | 2\|135 | 88\|9228 | 0.369918490542507 | 0.832362184316392 | ENSG00000089685 ENSG00000171791 | BIRC5 BCL2 | hsa:332 hsa:596 |
| hsa05203 | Viral carcinogenesis | 5\|135 | 275\|9228 | 0.375713108005613 | 0.832362184316392 | ENSG00000179388 ENSG00000117399 ENSG00000124635 ENSG00000145386 novel.600 | EGR3 CDC20 HIST1H2BJ CCNA2 - | hsa:1960 hsa:991 hsa:8970 hsa:890 hsa:1388 |
| hsa00983 | Drug metabolism - other enzymes | 2\|135 | 91\|9228 | 0.385627960985994 | 0.832362184316392 | ENSG00000167900 ENSG00000128951 | TK1 DUT | hsa:7083 hsa:1854 |
| hsa04659 | Th17 cell differentiation | 2\|135 | 91\|9228 | 0.385627960985994 | 0.832362184316392 | ENSG00000131759 ENSG00000104998 | RARA IL27RA | hsa:5914 hsa:9466 |
| hsa01040 | Biosynthesis of unsaturated fatty acids | 1\|135 | 33\|9228 | 0.385648145441102 | 0.832362184316392 | ENSG00000097021 | ACOT7 | hsa:11332 |
| hsa00410 | beta-Alanine metabolism | 1\|135 | 34\|9228 | 0.394667993224185 | 0.832362184316392 | ENSG00000137124 | ALDH1B1 | hsa:219 |
| hsa04261 | Adrenergic signaling in cardiomyocytes | 3\|135 | 155\|9228 | 0.39643388641381 | 0.832362184316392 | ENSG00000171791 ENSG00000164742 novel.600 | BCL2 ADCY1 - | hsa:596 hsa:107 hsa:1388 |
| hsa04713 | Circadian entrainment | 2\|135 | 94\|9228 | 0.401168157657756 | 0.832362184316392 | ENSG00000127588 ENSG00000164742 | GNG13 ADCY1 | hsa:51764 hsa:107 |
| hsa05146 | Amoebiasis | 2\|135 | 94\|9228 | 0.401168157657756 | 0.832362184316392 | ENSG00000164742 ENSG00000168542 | ADCY1 COL3A1 | hsa:107 hsa:1281 |
| hsa05212 | Pancreatic cancer | 2\|135 | 96\|9228 | 0.411426898319464 | 0.832362184316392 | novel.507 ENSG00000101412 | - E2F1 | hsa:1870 hsa:1869 |
| hsa00062 | Fatty acid elongation | 1\|135 | 36\|9228 | 0.412315205378326 | 0.832362184316392 | ENSG00000097021 | ACOT7 | hsa:11332 |
| hsa03020 | RNA polymerase | 1\|135 | 36\|9228 | 0.412315205378326 | 0.832362184316392 | ENSG00000105258 | POLR2I | hsa:5438 |
| hsa04120 | Ubiquitin mediated proteolysis | 4\|135 | 224\|9228 | 0.415734975972712 | 0.832362184316392 | ENSG00000224126 ENSG00000117399 ENSG00000108106 ENSG00000224080 | UBE2SP2 CDC20 UBE2S UBE2FP1 | hsa:27338 hsa:991 hsa:27338 hsa:140739 |
| hsa04064 | NF-kappa B signaling pathway | 2\|135 | 98\|9228 | 0.421599841169086 | 0.832362184316392 | ENSG00000107562 ENSG00000171791 | CXCL12 BCL2 | hsa:6387 hsa:596 |
| hsa05230 | Central carbon metabolism in cancer | 2\|135 | 98\|9228 | 0.421599841169086 | 0.832362184316392 | ENSG00000141526 ENSG00000117394 | SLC16A3 SLC2A1 | hsa:9123 hsa:6513 |
| hsa05220 | Chronic myeloid leukemia | 2\|135 | 100\|9228 | 0.431683304991233 | 0.832362184316392 | novel.507 ENSG00000101412 | - E2F1 | hsa:1870 hsa:1869 |
| hsa00051 | Fructose and mannose metabolism | 1\|135 | 39\|9228 | 0.43783293663354 | 0.832362184316392 | ENSG00000138030 | KHK | hsa:3795 |
| hsa00350 | Tyrosine metabolism | 1\|135 | 39\|9228 | 0.43783293663354 | 0.832362184316392 | ENSG00000180176 | TH | hsa:7054 |
| hsa04723 | Retrograde endocannabinoid signaling | 3\|135 | 166\|9228 | 0.439516104644506 | 0.832362184316392 | ENSG00000127588 ENSG00000187730 ENSG00000164742 | GNG13 GABRD ADCY1 | hsa:51764 hsa:2563 hsa:107 |
| hsa04933 | AGE-RAGE signaling pathway in diabetic complications | 2\|135 | 102\|9228 | 0.441673891202143 | 0.832362184316392 | ENSG00000171791 ENSG00000168542 | BCL2 COL3A1 | hsa:596 hsa:1281 |
| hsa04211 | Longevity regulating pathway | 2\|135 | 103\|9228 | 0.446633367194161 | 0.832362184316392 | ENSG00000164742 novel.600 | ADCY1 - | hsa:107 hsa:1388 |
| hsa04925 | Aldosterone synthesis and secretion | 2\|135 | 103\|9228 | 0.446633367194161 | 0.832362184316392 | ENSG00000164742 novel.600 | ADCY1 - | hsa:107 hsa:1388 |
| hsa04724 | Glutamatergic synapse | 2\|135 | 106\|9228 | 0.461364171539933 | 0.852068965456633 | ENSG00000127588 ENSG00000164742 | GNG13 ADCY1 | hsa:51764 hsa:107 |
| hsa05210 | Colorectal cancer | 2\|135 | 108\|9228 | 0.471058366324988 | 0.859724663548459 | ENSG00000089685 ENSG00000171791 | BIRC5 BCL2 | hsa:332 hsa:596 |
| hsa04062 | Chemokine signaling pathway | 3\|135 | 175\|9228 | 0.473897009663297 | 0.859724663548459 | ENSG00000107562 ENSG00000127588 ENSG00000164742 | CXCL12 GNG13 ADCY1 | hsa:6387 hsa:51764 hsa:107 |
| hsa05152 | Tuberculosis | 3\|135 | 178\|9228 | 0.485148931967658 | 0.86411365633467 | ENSG00000171791 ENSG00000102879 ENSG00000187796 | BCL2 CORO1A CARD9 | hsa:596 hsa:11151 hsa:64170 |
| hsa04625 | C-type lectin receptor signaling pathway | 2\|135 | 111\|9228 | 0.485404158908245 | 0.86411365633467 | ENSG00000179388 ENSG00000187796 | EGR3 CARD9 | hsa:1960 hsa:64170 |
| hsa05414 | Dilated cardiomyopathy (DCM) | 2\|135 | 112\|9228 | 0.490132884001145 | 0.86411365633467 | ENSG00000005961 ENSG00000164742 | ITGA2B ADCY1 | hsa:3674 hsa:107 |
| hsa00620 | Pyruvate metabolism | 1\|135 | 46\|9228 | 0.493177062395885 | 0.86411365633467 | ENSG00000137124 | ALDH1B1 | hsa:219 |
| hsa04979 | Cholesterol metabolism | 1\|135 | 47\|9228 | 0.500628717435806 | 0.869736331138476 | ENSG00000167780 | SOAT2 | hsa:8435 |
| hsa04210 | Apoptosis | 3\|135 | 185\|9228 | 0.510951293184738 | 0.880145845957848 | ENSG00000123416 ENSG00000089685 ENSG00000171791 | TUBA1B BIRC5 BCL2 | hsa:10376 hsa:332 hsa:596 |
| hsa00071 | Fatty acid degradation | 1\|135 | 49\|9228 | 0.515207324463131 | 0.880145845957848 | ENSG00000137124 | ALDH1B1 | hsa:219 |
| hsa02010 | ABC transporters | 1\|135 | 51\|9228 | 0.529363385850476 | 0.889504049994653 | ENSG00000144452 | ABCA12 | hsa:26154 |
| hsa04913 | Ovarian steroidogenesis | 1\|135 | 51\|9228 | 0.529363385850476 | 0.889504049994653 | ENSG00000164742 | ADCY1 | hsa:107 |
| hsa04935 | Growth hormone synthesis, secretion and action | 2\|135 | 122\|9228 | 0.535901135845306 | 0.893168559742176 | ENSG00000164742 novel.600 | ADCY1 - | hsa:107 hsa:1388 |
| hsa00380 | Tryptophan metabolism | 1\|135 | 54\|9228 | 0.549831705864447 | 0.908995965340417 | ENSG00000137124 | ALDH1B1 | hsa:219 |
| hsa04530 | Tight junction | 3\|135 | 200\|9228 | 0.563876557435676 | 0.920394635533783 | ENSG00000123416 ENSG00000166025 ENSG00000109062 | TUBA1B AMOTL1 SLC9A3R1 | hsa:10376 hsa:154810 hsa:9368 |
| hsa04340 | Hedgehog signaling pathway | 1\|135 | 58\|9228 | 0.575754481855909 | 0.920394635533783 | ENSG00000171791 | BCL2 | hsa:596 |
| hsa05130 | Pathogenic Escherichia coli infection | 4\|135 | 276\|9228 | 0.578375007912651 | 0.920394635533783 | ENSG00000214222 ENSG00000123416 ENSG00000109062 ENSG00000104833 | TUBBP2 TUBA1B SLC9A3R1 TUBB4A | hsa:203068 hsa:10376 hsa:9368 hsa:10382 |
| hsa05225 | Hepatocellular carcinoma | 3\|135 | 205\|9228 | 0.580740010967768 | 0.920394635533783 | novel.507 ENSG00000101412 ENSG00000164362 | - E2F1 TERT | hsa:1870 hsa:1869 hsa:7015 |
| hsa00280 | Valine, leucine and isoleucine degradation | 1\|135 | 59\|9228 | 0.582000190138292 | 0.920394635533783 | ENSG00000137124 | ALDH1B1 | hsa:219 |
| hsa04022 | cGMP-PKG signaling pathway | 3\|135 | 207\|9228 | 0.587370959087206 | 0.920394635533783 | ENSG00000164742 novel.600 ENSG00000121361 | ADCY1 - KCNJ8 | hsa:107 hsa:1388 hsa:3764 |
| hsa04330 | Notch signaling pathway | 1\|135 | 60\|9228 | 0.588154620755734 | 0.920394635533783 | ENSG00000135547 | HEY2 | hsa:23493 |
| hsa04061 | Viral protein interaction with cytokine and cytokine receptor | 1\|135 | 61\|9228 | 0.594219097871569 | 0.922840265633877 | ENSG00000107562 | CXCL12 | hsa:6387 |
| hsa05014 | Amyotrophic lateral sclerosis (ALS) | 1\|135 | 62\|9228 | 0.600194926581871 | 0.925112480821681 | ENSG00000171791 | BCL2 | hsa:596 |
| hsa04024 | cAMP signaling pathway | 3\|135 | 214\|9228 | 0.610052674103601 | 0.933289538740583 | ENSG00000164128 ENSG00000168539 ENSG00000164742 | NPY1R CHRM1 ADCY1 | hsa:4886 hsa:1128 hsa:107 |
| hsa04550 | Signaling pathways regulating pluripotency of stem cells | 2\|135 | 144\|9228 | 0.626392608137479 | 0.940077587393366 | ENSG00000160867 ENSG00000130829 | FGFR4 DUSP9 | hsa:2264 hsa:1852 |
| hsa05167 | Kaposi sarcoma-associated herpesvirus infection | 3\|135 | 220\|9228 | 0.628830021161235 | 0.940077587393366 | novel.507 ENSG00000127588 ENSG00000101412 | - GNG13 E2F1 | hsa:1870 hsa:51764 hsa:1869 |
| hsa04722 | Neurotrophin signaling pathway | 2\|135 | 145\|9228 | 0.630166365741186 | 0.940077587393366 | ENSG00000007264 ENSG00000171791 | MATK BCL2 | hsa:4145 hsa:596 |
| hsa04270 | Vascular smooth muscle contraction | 2\|135 | 146\|9228 | 0.633910688109447 | 0.940077587393366 | ENSG00000122679 ENSG00000164742 | RAMP3 ADCY1 | hsa:10268 hsa:107 |
| hsa04217 | Necroptosis | 3\|135 | 223\|9228 | 0.637985353494568 | 0.940077587393366 | ENSG00000184260 ENSG00000171791 ENSG00000218690 | HIST2H2AC BCL2 HIST1H2APS4 | hsa:8338 hsa:596 hsa:8336 |
| hsa04610 | Complement and coagulation cascades | 1\|135 | 71\|9228 | 0.650194453462432 | 0.940077587393366 | ENSG00000131187 | F12 | hsa:2161 |
| hsa04920 | Adipocytokine signaling pathway | 1\|135 | 71\|9228 | 0.650194453462432 | 0.940077587393366 | ENSG00000117394 | SLC2A1 | hsa:6513 |
| hsa04720 | Long-term potentiation | 1\|135 | 72\|9228 | 0.655351573565366 | 0.940077587393366 | ENSG00000164742 | ADCY1 | hsa:107 |
| hsa04066 | HIF-1 signaling pathway | 2\|135 | 152\|9228 | 0.655761439010982 | 0.940077587393366 | ENSG00000171791 ENSG00000117394 | BCL2 SLC2A1 | hsa:596 hsa:6513 |
| hsa04978 | Mineral absorption | 1\|135 | 73\|9228 | 0.660433218122889 | 0.940200067466613 | ENSG00000198417 | MT1F | hsa:4494 |
| hsa05323 | Rheumatoid arthritis | 1\|135 | 75\|9228 | 0.670374450698844 | 0.945055465842408 | ENSG00000107562 | CXCL12 | hsa:6387 |
| hsa04213 | Longevity regulating pathway - multiple species | 1\|135 | 77\|9228 | 0.680026734627953 | 0.945055465842408 | ENSG00000164742 | ADCY1 | hsa:107 |
| hsa04371 | Apelin signaling pathway | 2\|135 | 161\|9228 | 0.686585906909477 | 0.945055465842408 | ENSG00000127588 ENSG00000164742 | GNG13 ADCY1 | hsa:51764 hsa:107 |
| hsa05170 | Human immunodeficiency virus 1 infection | 3\|135 | 242\|9228 | 0.69231130146639 | 0.945055465842408 | ENSG00000127588 ENSG00000171791 ENSG00000157456 | GNG13 BCL2 CCNB2 | hsa:51764 hsa:596 hsa:9133 |
| hsa03320 | PPAR signaling pathway | 1\|135 | 81\|9228 | 0.698497568217545 | 0.945055465842408 | ENSG00000214456 | PLIN5 | hsa:440503 |
| hsa04068 | FoxO signaling pathway | 2\|135 | 165\|9228 | 0.699548831934128 | 0.945055465842408 | ENSG00000166851 ENSG00000157456 | PLK1 CCNB2 | hsa:5347 hsa:9133 |
| hsa03013 | RNA transport | 3\|135 | 246\|9228 | 0.702941875975185 | 0.945055465842408 | novel.674 ENSG00000013810 ENSG00000183684 | - TACC3 ALYREF | hsa:79897 hsa:10460 hsa:10189 |
| hsa04510 | Focal adhesion | 3\|135 | 249\|9228 | 0.710731834700372 | 0.945055465842408 | ENSG00000005961 ENSG00000171791 ENSG00000037280 | ITGA2B BCL2 FLT4 | hsa:3674 hsa:596 hsa:2324 |
| hsa05160 | Hepatitis C | 2\|135 | 173\|9228 | 0.724151941925675 | 0.945055465842408 | novel.507 ENSG00000101412 | - E2F1 | hsa:1870 hsa:1869 |
| hsa04971 | Gastric acid secretion | 1\|135 | 88\|9228 | 0.72831015636435 | 0.945055465842408 | ENSG00000164742 | ADCY1 | hsa:107 |
| hsa05211 | Renal cell carcinoma | 1\|135 | 90\|9228 | 0.736277165891113 | 0.945055465842408 | ENSG00000117394 | SLC2A1 | hsa:6513 |
| hsa04512 | ECM-receptor interaction | 1\|135 | 91\|9228 | 0.740173268167837 | 0.945055465842408 | ENSG00000005961 | ITGA2B | hsa:3674 |
| hsa04137 | Mitophagy - animal | 2\|135 | 180\|9228 | 0.744275780158218 | 0.945055465842408 | ENSG00000254337 ENSG00000101412 | AC083967.1 E2F1 | hsa:55626 hsa:1869 |
| hsa05142 | Chagas disease (American trypanosomiasis) | 1\|135 | 93\|9228 | 0.747794887976192 | 0.945055465842408 | ENSG00000164742 | ADCY1 | hsa:107 |
| hsa04972 | Pancreatic secretion | 1\|135 | 94\|9228 | 0.751522057119401 | 0.945055465842408 | ENSG00000164742 | ADCY1 | hsa:107 |
| hsa04014 | Ras signaling pathway | 3\|135 | 269\|9228 | 0.758724779844473 | 0.945055465842408 | ENSG00000160867 ENSG00000127588 ENSG00000037280 | FGFR4 GNG13 FLT4 | hsa:2264 hsa:51764 hsa:2324 |
| hsa04750 | Inflammatory mediator regulation of TRP channels | 1\|135 | 96\|9228 | 0.758813153797183 | 0.945055465842408 | ENSG00000164742 | ADCY1 | hsa:107 |
| hsa01521 | EGFR tyrosine kinase inhibitor resistance | 1\|135 | 97\|9228 | 0.76237866236457 | 0.945055465842408 | ENSG00000171791 | BCL2 | hsa:596 |
| hsa05418 | Fluid shear stress and atherosclerosis | 2\|135 | 188\|9228 | 0.765734902044079 | 0.945055465842408 | ENSG00000005961 ENSG00000171791 | ITGA2B BCL2 | hsa:3674 hsa:596 |
| hsa04916 | Melanogenesis | 1\|135 | 102\|9228 | 0.779436412867484 | 0.945055465842408 | ENSG00000164742 | ADCY1 | hsa:107 |
| hsa04726 | Serotonergic synapse | 1\|135 | 103\|9228 | 0.782699187825066 | 0.945055465842408 | ENSG00000127588 | GNG13 | hsa:51764 |
| hsa04621 | NOD-like receptor signaling pathway | 2\|135 | 195\|9228 | 0.783222563116822 | 0.945055465842408 | ENSG00000171791 ENSG00000187796 | BCL2 CARD9 | hsa:596 hsa:64170 |
| hsa00010 | Glycolysis / Gluconeogenesis | 1\|135 | 104\|9228 | 0.785914049155873 | 0.945055465842408 | ENSG00000137124 | ALDH1B1 | hsa:219 |
| hsa04912 | GnRH signaling pathway | 1\|135 | 104\|9228 | 0.785914049155873 | 0.945055465842408 | ENSG00000164742 | ADCY1 | hsa:107 |
| hsa03018 | RNA degradation | 1\|135 | 105\|9228 | 0.789081695293966 | 0.945055465842408 | ENSG00000130520 | LSM4 | hsa:25804 |
| hsa05410 | Hypertrophic cardiomyopathy (HCM) | 1\|135 | 106\|9228 | 0.792202814567813 | 0.945055465842408 | ENSG00000005961 | ITGA2B | hsa:3674 |
| hsa00564 | Glycerophospholipid metabolism | 1\|135 | 107\|9228 | 0.795278085345422 | 0.945055465842408 | ENSG00000143845 | ETNK2 | hsa:55224 |
| hsa05412 | Arrhythmogenic right ventricular cardiomyopathy (ARVC) | 1\|135 | 107\|9228 | 0.795278085345422 | 0.945055465842408 | ENSG00000005961 | ITGA2B | hsa:3674 |
| hsa04020 | Calcium signaling pathway | 2\|135 | 202\|9228 | 0.799563407198415 | 0.945055465842408 | ENSG00000168539 ENSG00000164742 | CHRM1 ADCY1 | hsa:1128 hsa:107 |
| hsa05202 | Transcriptional misregulation in cancer | 2\|135 | 205\|9228 | 0.806229139060673 | 0.945055465842408 | ENSG00000131759 ENSG00000145386 | RARA CCNA2 | hsa:5914 hsa:890 |
| hsa05100 | Bacterial invasion of epithelial cells | 1\|135 | 112\|9228 | 0.809989775292436 | 0.945055465842408 | ENSG00000116670 | MAD2L2 | hsa:10459 |
| hsa04060 | Cytokine-cytokine receptor interaction | 2\|135 | 208\|9228 | 0.812698654525466 | 0.945055465842408 | ENSG00000107562 ENSG00000104998 | CXCL12 IL27RA | hsa:6387 hsa:9466 |
| hsa04141 | Protein processing in endoplasmic reticulum | 2\|135 | 210\|9228 | 0.816904804257254 | 0.945055465842408 | ENSG00000171791 novel.600 | BCL2 - | hsa:596 hsa:1388 |
| hsa04931 | Insulin resistance | 1\|135 | 116\|9228 | 0.820999520967843 | 0.945055465842408 | ENSG00000117394 | SLC2A1 | hsa:6513 |
| hsa04740 | Olfactory transduction | 1\|135 | 123\|9228 | 0.838764104929712 | 0.945055465842408 | ENSG00000127588 | GNG13 | hsa:51764 |
| hsa04922 | Glucagon signaling pathway | 1\|135 | 123\|9228 | 0.838764104929712 | 0.945055465842408 | ENSG00000117394 | SLC2A1 | hsa:6513 |
| hsa05145 | Toxoplasmosis | 1\|135 | 125\|9228 | 0.843510212531183 | 0.945055465842408 | ENSG00000171791 | BCL2 | hsa:596 |
| hsa03015 | mRNA surveillance pathway | 1\|135 | 126\|9228 | 0.845830999228787 | 0.945055465842408 | ENSG00000183684 | ALYREF | hsa:10189 |
| hsa04668 | TNF signaling pathway | 1\|135 | 126\|9228 | 0.845830999228787 | 0.945055465842408 | novel.600 | - | hsa:1388 |
| hsa04514 | Cell adhesion molecules (CAMs) | 1\|135 | 128\|9228 | 0.850370571791171 | 0.945055465842408 | ENSG00000105695 | MAG | hsa:4099 |
| hsa03040 | Spliceosome | 2\|135 | 229\|9228 | 0.85285493258949 | 0.945055465842408 | ENSG00000130520 ENSG00000183684 | LSM4 ALYREF | hsa:25804 hsa:10189 |
| hsa04152 | AMPK signaling pathway | 1\|135 | 134\|9228 | 0.86320833319823 | 0.951385528524931 | ENSG00000145386 | CCNA2 | hsa:890 |
| hsa04010 | MAPK signaling pathway | 3\|135 | 331\|9228 | 0.868085720040814 | 0.951644773306775 | ENSG00000160867 ENSG00000130829 ENSG00000037280 | FGFR4 DUSP9 FLT4 | hsa:2264 hsa:1852 hsa:2324 |
| hsa05012 | Parkinson disease | 4\|135 | 430\|9228 | 0.880695553944259 | 0.954234887221622 | ENSG00000214222 ENSG00000123416 ENSG00000180176 ENSG00000104833 | TUBBP2 TUBA1B TH TUBB4A | hsa:203068 hsa:10376 hsa:7054 hsa:10382 |
| hsa05162 | Measles | 1\|135 | 144\|9228 | 0.882219578824601 | 0.954234887221622 | ENSG00000171791 | BCL2 | hsa:596 |
| hsa04670 | Leukocyte transendothelial migration | 1\|135 | 147\|9228 | 0.887393596851665 | 0.954234887221622 | ENSG00000107562 | CXCL12 | hsa:6387 |
| hsa04919 | Thyroid hormone signaling pathway | 1\|135 | 148\|9228 | 0.889067626630877 | 0.954234887221622 | ENSG00000117394 | SLC2A1 | hsa:6513 |
| hsa00230 | Purine metabolism | 1\|135 | 153\|9228 | 0.897074362494444 | 0.957813772455005 | ENSG00000164742 | ADCY1 | hsa:107 |
| hsa04140 | Autophagy - animal | 2\|135 | 270\|9228 | 0.909594715516697 | 0.960899270256224 | ENSG00000254337 ENSG00000171791 | AC083967.1 BCL2 | hsa:55626 hsa:596 |
| hsa04142 | Lysosome | 1\|135 | 162\|9228 | 0.910067741676263 | 0.960899270256224 | ENSG00000102393 | GLA | hsa:2717 |
| hsa04072 | Phospholipase D signaling pathway | 1\|135 | 165\|9228 | 0.914026135121774 | 0.960899270256224 | ENSG00000164742 | ADCY1 | hsa:107 |
| hsa04310 | Wnt signaling pathway | 1\|135 | 169\|9228 | 0.91903619536865 | 0.961236836992721 | ENSG00000175792 | RUVBL1 | hsa:8607 |
| hsa04921 | Oxytocin signaling pathway | 1\|135 | 180\|9228 | 0.931368528461804 | 0.967429458618471 | ENSG00000164742 | ADCY1 | hsa:107 |
| hsa01200 | Carbon metabolism | 1\|135 | 183\|9228 | 0.934395281982718 | 0.967429458618471 | ENSG00000092621 | PHGDH | hsa:26227 |
| hsa04390 | Hippo signaling pathway | 1\|135 | 193\|9228 | 0.943559137599645 | 0.972008156823755 | ENSG00000089685 | BIRC5 | hsa:332 |
| hsa05132 | Salmonella infection | 2\|135 | 317\|9228 | 0.949380363990621 | 0.973114873090386 | ENSG00000171791 ENSG00000233328 | BCL2 PFN1P1 | hsa:596 hsa:5216 |
| hsa04144 | Endocytosis | 2\|135 | 343\|9228 | 0.963576772120136 | 0.980663211172639 | ENSG00000160867 ENSG00000024422 | FGFR4 EHD2 | hsa:2264 hsa:30846 |
| hsa05205 | Proteoglycans in cancer | 1\|135 | 238\|9228 | 0.971381118768014 | 0.980663211172639 | ENSG00000090447 | TFAP4 | hsa:7023 |
| hsa05017 | Spinocerebellar ataxia | 1\|135 | 240\|9228 | 0.972234233738901 | 0.980663211172639 | ENSG00000254337 | AC083967.1 | hsa:55626 |
| hsa05131 | Shigellosis | 2\|135 | 375\|9228 | 0.975879488191309 | 0.980663211172639 | ENSG00000171791 ENSG00000233328 | BCL2 PFN1P1 | hsa:596 hsa:5216 |
| hsa04714 | Thermogenesis | 1\|135 | 360\|9228 | 0.995538413440287 | 0.995538413440287 | ENSG00000164742 | ADCY1 | hsa:107 |
